# Metabolomics of Cerebrospinal Fluid from Healthy Subjects Reveal Metabolites Associated with Ageing

**DOI:** 10.3390/metabo11020126

**Published:** 2021-02-23

**Authors:** Henrik Carlsson, Niclas Rollborn, Stephanie Herman, Eva Freyhult, Anders Svenningsson, Joachim Burman, Kim Kultima

**Affiliations:** 1 Department of Medical Sciences, Clinical Chemistry, Uppsala University, 751 85 Uppsala, Sweden; henrik.carlsson@medsci.uu.se (H.C.); niclas.rollborn@medsci.uu.se (N.R.); stephanie.herman@medsci.uu.se (S.H.); eva.freyhult@medsci.uu.se (E.F.); 2 Department of Clinical Sciences, Karolinska Institutet, Danderyd Hospital, 182 88 Stockholm, Sweden; anders.svenningsson@ki.se; 3 Department of Neuroscience, Uppsala University, 751 85 Uppsala, Sweden; joachim.burman@neuro.uu.se

**Keywords:** metabolomics, cerebrospinal fluid, ageing, high-resolution mass spectrometry

## Abstract

To increase our understanding of age-related diseases affecting the central nervous system (CNS) it is important to understand the molecular processes of biological ageing. Metabolomics of cerebrospinal fluid (CSF) is a promising methodology to increase our understanding of naturally occurring processes of ageing of the brain and CNS that could be reflected in CSF. In the present study the CSF metabolomes of healthy subjects aged 30–74 years (*n* = 23) were studied using liquid chromatography high-resolution mass spectrometry (LC-HRMS), and investigated in relation to age. Ten metabolites were identified with high confidence as significantly associated with ageing, eight with increasing levels with ageing: isoleucine, acetylcarnitine, pipecolate, methionine, glutarylcarnitine, 5-hydroxytryptophan, ketoleucine, and hippurate; and two decreasing with ageing: methylthioadenosine and 3-methyladenine. To our knowledge, this is the first time the CSF metabolomes of healthy subjects are assessed in relation to ageing. The present study contributes to the field of ageing metabolomics by presenting a number of metabolites present in CSF with potential relevance for ageing and the results motivate further studies.

## 1. Introduction

Ageing is the single greatest risk factor associated with numerous diseases including cancer, metabolic and cardiac disease and diseases affecting the central nervous system (CNS) such as Alzheimer’s disease [1,2]. The population of virtually every country is continuously getting older and age-related diseases will be an increasing problem as well as becoming a greater burden to healthcare systems worldwide [3,4,5]. It is of great interest to find methods to allow the measurement of biological age, as opposed to chronological age, in order to treat and possibly counteract age-related diseases [6]. To allow the measurement of biological age, reliable biomarkers of ageing are required. Several such biomarkers have been suggested over the years, and although there is a consensus regarding the ‘hallmarks of ageing’ [7] there is still a need for robust ageing biomarkers [8,9]. Metabolomics allows for discovery-based experiments that survey the totality of metabolites in the studied biospecimen and is an optimal methodology for the discovery of novel biomarkers [10]. Metabolomic studies of blood collections (serum or plasma) [11,12,13,14,15,16,17,18,19] and urine [20] have previously demonstrated that metabolite levels are influenced by ageing, and metabolic drift as a result of ageing has also been demonstrated in different parts of the ageing murine brain [21].

The majority of studies on ageing and metabolomics focus on the blood metabolome. Regarding the ageing of the CNS, cerebrospinal fluid (CSF) is a better suited biospecimen due to its proximity to the CNS. CSF is interacting closely with CNS tissue and its composition can mirror changes occurring in the CNS and more than 450 metabolites have been identified in human CSF [22]. CSF metabolomics have been used to study alterations in the metabolome associated with several neurological diseases, e.g., Alzheimer’s disease [23], Parkinson’s disease [24], Huntington’s disease [25] and multiple sclerosis [26,27,28]. To our knowledge, ageing has only been assessed in relation to the CSF metabolome once previously [29]. This was a study in which HIV positive patients were compared with HIV negative controls (‘young’ and ‘old’) and the results suggested that the HIV positive patients exhibited accelerated ageing, since their CSF metabolomes overlapped with the controls of advanced age.

In the present study we measured the CSF metabolome of healthy subjects aged 30–74 years using liquid chromatography high-resolution mass spectrometry (LC-HRMS), and investigated their metabolomes in relation to ageing. The present study contributes to the field of ageing metabolomics by presenting a number of metabolites present in CSF with potential relevance for ageing. The fact that the participants are healthy makes the study unique, since CSF is rarely collected from healthy individuals, and allows for an assessment of ageing with a minimum of confounding factors.

## 2. Results

**Figure 1:**
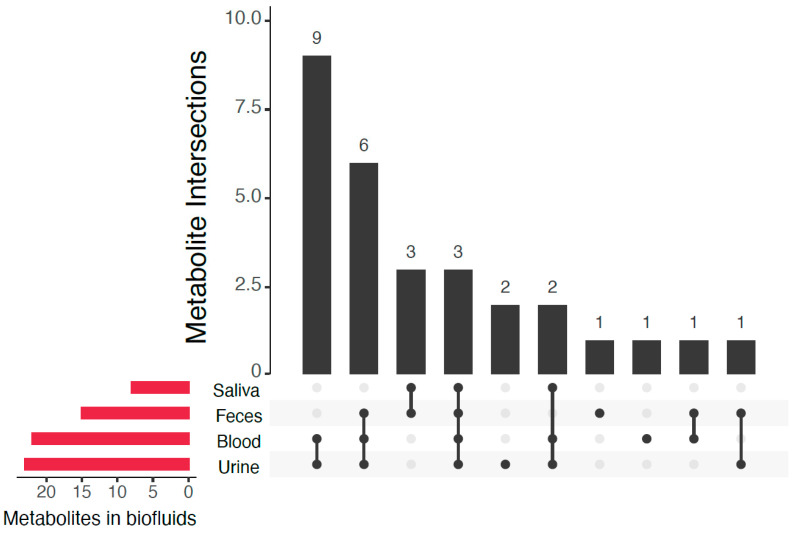
Intersections of biofluids in which the 31 metabolites previously not reported as CSF metabolites in the HMDB have been detected (based on HMDB data).

We investigated the effect of ageing in healthy individuals aged 30–74 years using LC-HRMS metabolomics. Using an in-house library of well characterized metabolites as reference we identified, with high-confidence, 70 metabolites in the CSF of these subjects (Supplementary Materials Table S1). Thirty-one of the detected metabolites are not listed as CSF metabolites in the CSF Metabolome database (part of the Human Metabolome Database, HMDB) [22], Table 1). However, several of them have been reported in CSF in the literature. We performed a literature review of these 31 metabolites and found 11 of them previously reported in CSF (Table 1), several of them reported by us when using the very same analytical method [27]. According to the HMDB, the majority of these 31 metabolites have previously been detected in either urine or blood (74% and 71% respectively, with an overlap of 80% between the two biofluids) and some of them have also been detected in feces and saliva (Table 1). An overview of the biofluids in which they have been previously observed is presented in Figure 1.

**Figure 2:**
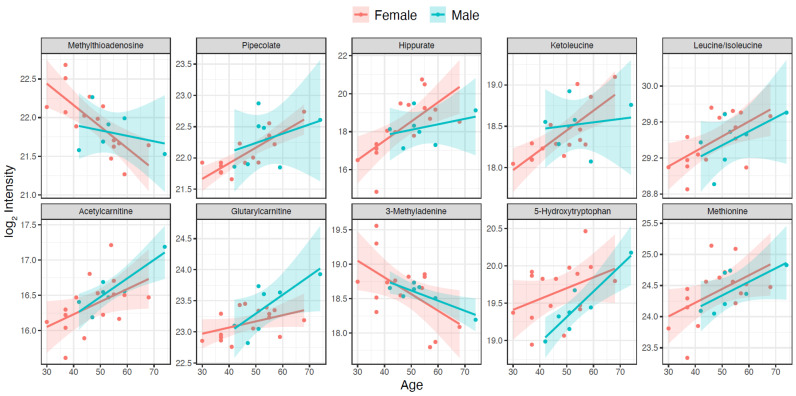
Metabolites significantly associated with age (q < 0.1) in CSF from healthy subjects. Value refers to log_2_ transformed peak areas and age is given in years. The colored zones refers to 95% confidence intervals of the regressions.

The association between metabolite levels and age was investigated using linear regression adjusting for gender and the interaction age:gender for the 70 identified metabolites. Correcting for multiple testing, we found ten metabolites significantly associated (q < 0.1) with age, and the single metabolite 5-hydroxytryptophan to be associated with both age and gender (Table 2 and Figure 2). Of the metabolites associated with age, the majority (8 of 10) were found to increase in level with increasing age: isoleucine, acetylcarnitine, pipecolate, methionine, glutarylcarnitine, 5-hydroxytryptophan, ketoleucine, and hippurate. Methylthioadenosine and 3-methyladenine were found to decrease with increasing age.

## 3. Discussion

In the present study we performed metabolomics of CSF from healthy subjects aged 30–74 years using LC-HRMS. We identified 70 metabolites, whereof 31 were not listed as CSF metabolites in the HMDB, but eleven of these 31 had previously been described in CSF in the literature by us and others. The majority of these have been reported to be present in either urine, blood, feces or saliva. One metabolite, 5-methylcytosine, has not been previously reported in human biofluids in HMDB or elsewhere to our knowledge. Probable sources of 5-methylcytosine are either food or endogenous formation. We found 10 metabolites significantly associated with age and one also with the interaction age:gender (*p* < 0.05).

To our knowledge this is the first time CSF from healthy subjects have been assessed with regard to ageing. As stated by the American Federation for Aging Research [40] in their criteria for biomarkers of ageing, such a biomarker should monitor a basic process that underlies the ageing process, not the effects of disease. Thus, it is of the utmost importance that when studying ageing using an explorative method such as metabolomics that the samples used are from healthy individuals. We want to acknowledge the complex nature of ageing, and it is to be expected that other factors, such as socio-economic circumstances, diet and exercise regimes, could affect the metabolome in similar ways as ageing. Ideally such data should be collected for the subjects in an ageing study. Due to regional ethical restrictions only age and gender data were collected for the healthy subjects in the present study, and no assessment of neurodegenerative or other CNS associated diseases were made prior to sample collection. Especially when investigating relatively small number of samples such limited subject data could have an effect on the observations of the study. The statistical analysis in the present study was performed with awareness of such risks, and therefore performed with adequate restrictions and correcting for multiple testing. To fully study the effect of ageing, longitudinal samples would also be required [11] but this was not possible within the scope of this small study. Our observations are in line with previous observations regarding metabolite levels and ageing, adding further strength to the results. Several of the metabolites found to be associated with age in the present study have previously been discussed in the context of ageing metabolomics and are described in the following.

Among amino acids, two of the most interesting are methionine and isoleucine. Methionine restriction has been shown to extend lifespan in rodents [41] and Drosophila [42], and it has been suggested that methionine has an essential role in the lifespan extension observed for various organisms when undergoing dietary restriction, regardless of total calorie intake [43]. In line with this, Johnson et al. reported that higher plasma levels of circulating methionine was associated with faster rate of biological ageing in humans [19]. Increased levels of isoleucine have previously been observed in aged rat brains [44], and the present results with increasing CSF levels with age indicate a similar situation in the human brain. Isoleucine has also been observed to increase with age in plasma in studies on metabolomics and age [13,17].

In connection with amino acids, another interesting metabolite is 5-hydroxy-l-tryptophan (also observed here with elevated levels in female subjects), which is produced from the essential amino acid l-tryptophan. 5-hydroxy-l-tryptophan is the immediate precursor to serotonin, and can thus affect neural signalling. 5-hydroxy-l-tryptophan has been observed to accumulate in CSF in patients with aromatic l-amino acid decarboxylase deficiency [45]. Increased CSF levels have also been observed in patients with Parkinson’s disease and severe postural instability and gait disorders [46]. Due to the direct connection to serotonin, which is believed to have a role in the ageing of the brain [47], our observation of elevated levels of 5-hydroxy-l-tryptophan in CSF of ageing patients is interesting but needs to be confirmed in larger studies.

Among the ten metabolites associated with age, we also found two acylcarnitines: acetylcarnitine and glutarylcarnitine, both increasing with age. Acylcarnitines, also known as carnitine esters, have important neuroprotective, neuromodulatory, and neurotrophic properties [48] and their potential role in ageing is an interesting topic. Increased levels of acylcarnitines with age have been observed in plasma in several studies on metabolomics and age [11,15,16], and high plasma levels have been associated with type 2 diabetes and obesity [49]. Hippurate, the metabolite increasing most with age in the present study, has also been previously associated with ageing in metabolomics studies of plasma [12,17].

Two of the ten metabolites exhibited lower levels with increased age: methylthioadenosine and 3-methyladenine. The latter metabolite has not previously been reported in CSF, only in urine and blood. To our knowledge there are no previous reports on any of these metabolites as associated with ageing.

To our knowledge, ageing has only been studied in relation to the CSF metabolome once previously [29]. In that study the CSF metabolomes of HIV positive patients were compared with HIV negative controls of different ages. Thirty-four named and 12 unnamed (‘unknown’) metabolites were altered in the older (age ≥ 50, *n* = 23) controls as compared to the younger controls (<50 years, *n* = 23), including metabolites associated with neurotransmitter production (glutamate, homocarnosine, 3-(4-hydroxyphenyl)lactate), markers of glial activation (choline and arachidonate), oxidative products (5-oxoproline) and markers of oxidative stress (urate, hypoxanthine), and metabolic waste products (3-hydroxybutyrate (BHBA), 1,2-propanediol, phenylacetylglutamine, lactate). There is no overlap when comparing the metabolites significantly associated with age in the former ‘HIV’ study with the healthy subjects from the present study. One major difference between the two studies, is that in the present study age is treated as a continuous variable, whereas in the former study age was treated as a binary variable, i.e., ‘young’ (<50 years) and ‘old’ (≥50 years). Here we demonstrate that age could be treated as a continuous variable in order to observe changes over the whole age range studied. In the present study all included subjects are also defined as healthy, whereas in the former study all controls were simply ‘HIV’ negative and pre-screened to not exhibit factors associated with HIV-associated neurocognitive disorders.

Although metabolomics have been used to study ageing for more than a decade, it is complex to derive robust ageing biomarkers from metabolomic data. Regarding the ageing plasma metabolome, some conclusions have been made: levels of steroids, amino acids, fatty acids, and metabolites related to kidney function are associated with ageing [16]. These observations have been replicated in several larger studies and have improved the understanding of the ageing plasma metabolome [15]. Much less is known about the ageing CSF metabolome. When comparing the little that is presently known about the ageing CSF metabolome with the ageing plasma metabolome, one striking difference is the large number of metabolites associated with ageing in plasma. Several studies on ageing in plasma describe approximately half of all detected metabolites to be associated with age (e.g., 57% of 1097 metabolites tested by Darst et al.) [11,14,15]. These results suggest that ageing affects a broad range of metabolic pathways. Such a large influence of ageing on the metabolome was not observed in CSF in the present study. These experiments should be replicated on a larger set of samples using the same analytical platform and data-analyzing pipelines to compare the plasma and CSF metabolomes as regards to ageing, ideally comparing the different sample matrices in the same individuals.

The major limitation of the present study is the small sample size, 23 samples with an uneven gender distribution (16 female and 7 male samples), limiting the power of the statistical tests performed and thereby the strength of the biological findings. The small sample size is based on the fact that only healthy subjects were considered for the study, and due to the invasive nature of lumbar punctures, CSF is rarely collected from healthy individuals. Despite the small number of samples, the ‘health’ aspect makes the study unique and the results valuable with regard to ageing. The presented results provide insights regarding the constituents of CSF affected by ageing and add knowledge to a subject with limited previous research. Ageing will be a confounding influence in most metabolomic studies and it is of importance to establish the healthy ageing CSF metabolome. An improved understanding of the ageing metabolome will thus improve the interpretation of all metabolomic data from subjects of varying age.

In summary we identified ten CSF metabolites associated with participant age in a small but healthy cohort. The majority of the available data on these metabolites describe plasma and serum levels, and the CSF data presented herein adds a new and previously unexplored dimension as regards to ageing. The study indicates that the CSF metabolome is a promising avenue for assessing ageing of the CNS and the results herein motivate further studies.

## 4. Materials and Methods

### 4.1. Chemicals

Methanol (≥99.9%, MS grade) from Honeywell (Seelze, Germany), acetonitrile (≥99.9%, MS grade) and isopropanol (≥99.9%, MS grade) from VWR Chemicals (Leuven, Belgium), formic acid (American Chemical Society reagent grade) from Merck (Darmstadt, Germany). Water was purified using an Advantage A10 Milli-Q system (Merck Millipore, Burlington, MA, USA).

### 4.2. Ethical Approval

The study was approved by the Regional Ethical Review Board of Umeå, Sweden (Dnr 08-157M) with the 1964 Helsinki declaration and its later amendments or comparable ethical standards. All participants provided written informed consent before any samples were collected.

### 4.3. Study Cohort and Collection of Samples

Samples were collected at the University Hospital of Umeå by lumbar punctures from healthy individuals. The participants had, to their own and to the authors’ best knowledge, no ongoing disease. This is thus how ‘healthy’ was defined in the present study. Complete medical examinations were not made, nor any assessment for neuro-degenerative and other CNS associated diseases. Participant gender and age were recorded for use in the statistical evaluation, but no other information was collected due to regional ethical restrictions. The demographic data of the patients are summarized in Table 3. Detailed data of participants are presented in Table S2. The samples were stored at −80 °C until analysis.

### 4.4. Metabolite Extraction

Metabolite extraction of CSF metabolites and mass spectrometry analysis was done as previously described [26]. Briefly, CSF samples were thawed on ice and 100 µL was mixed with 410 µL ice-cold methanol (MeOH) supplemented with internal standards. The samples were vortexed for 15 s and incubated at −20 °C for 30 min, followed by centrifugation at 20,400× *g* for 12 min at 4 °C. The supernatants were transferred to fresh Eppendorf tubes which were dried overnight using a centrifugal vacuum concentrator. Upon analysis the dried samples were reconstituted in 100 µL 5% MeOH, 95 % H_2_O with 0.1% formic acid. 10 µL of each sample was pooled to create a quality control (QC) sample to be injected repeatedly to monitor the MS performance by ensuring that the internal standards were present and in correct magnitude.

### 4.5. Mass Spectrometry Analysis

Samples were injected in a constrained randomized order. A QC injection was done every 8th sample followed by a blank injection. Prior to the injection of samples, five repeated injections of the QC were done to precondition the column, followed by two blank injections. Following, a 2-fold serial dilution series ranging from 0.5 to 32.0 µL QC was injected. In addition, MS/MS analysis was performed on subpools of samples. The injection volume was 10 µL and a Thermo Accucore aQ RP C18 column (100 × 2.1 mm, 2.6 µm particle size, Thermo Fisher Scientific, Waltham, MA, USA) was used for the chromatography. The analysis was performed on a Thermo Ultimate 3000 HPLC and Thermo Q-Exactive Orbitrap mass spectrometer (Thermo Fisher Scientific). Details of the LC-HRMS analysis have been previously described [26].

### 4.6. Peak-Picking and Quality Assessment

Acquired raw data was converted to an open-source format (mzML) and peak-picked using *msconvert* from ProteoWizard (Palo Alto, CA, USA) [50]. Preprocessing was done using the OpenMS tools [51] within the KNIME platform [52]. Integrated peak areas were loaded into the statistical software environment R v3.6.0 [53]. Contaminants were detected and removed using the blank injections, according to our previously introduced pipeline [54], and by removing features whose peak areas in the dilution series did not achieve a statistically significant (*p*-value < 0.05) Pearson’s correlation with the injection volume of the series. The integrated peak areas were further log2 transformed and run order effects were corrected by fitting a LOESS curve for each metabolite using the function “loessFit” from the R-package limma and a span of 0.2, which were used for normalization [55]. Finally, only stable features with a coefficient of variance in the QC samples lower than 20% were kept.

### 4.7. Metabolite Identification

All metabolic features with a 75% coverage were matched against an in house library of characterized metabolites using a 15 ppm mass tolerance and a 20 s time window. The exact same setup was used for characterization of standards as for samples of interest. Metabolites (identified metabolic features) of interest were manually curated on MS/MS level when available. Identities were confirmed by matching m/z and elution time of the pure standards with the metabolite peaks and by comparing MS/MS fragmentation patterns. Identities of metabolites with available MS/MS fragmentation that did not match the authentic standard were rejected.

### 4.8. Statistical Analysis

The association between metabolite levels and age was investigated using linear regression with age, gender and the interaction age:gender as independent variables and metabolite levels as dependent variables. The age association was estimated using a global F-test evaluating the significance of age and age:gender. The False Discovery Adjusted p-values, q-values, with the limit q < 0.1 were used for the final selection of metabolites significantly associated with age. In post hoc tests, variables were evaluated individually with *p* < 0.05 as the limit for significance. 

## Figures and Tables

**Table 1:** CSF metabolites previously not reported in the Human Metabolome Database (HMDB).

Metabolite	HMDB ID	Formula	Mass (Da) ^1^	Reported in CSF in Literature ^2^	Previously Detected in (Biofluids) ^3^
Cortexolone (11-deoxycortisol)	HMDB0000015	C_21_H_30_O_4_	346.214	No	Urine, blood
1-Methyladenosine	HMDB0003331	C_11_H_15_N_5_O_4_	281.112	[27]	Urine, blood
3-(2-Hydroxyphenyl)propanoic acid (melilotic acid)	HMDB0033752	C_9_H_10_O_3_	166.063	No	Feces
3-Methyladenine	HMDB0011600	C_6_H_7_N_5_	149.070	No	Urine, blood
4-Acetamidobutanoic acid	HMDB0003681	C_6_H_11_NO_3_	145.074	[27,29]	Urine, blood, feces
4-Methylcatechol	HMDB0000873	C_7_H_8_O_2_	124.052	No	Urine, blood, feces
5-Methylcytosine	HMDB0002894	C_5_H_7_N_3_O	125.059	No	Not previously reported (possible source: food/endogenous)
Aldosterone	HMDB0000037	C_21_H_28_O_5_	360.194	No	Urine, blood, saliva
Aminoadipic acid	HMDB0000510	C_6_H_11_NO_4_	161.069	No	Urine, blood, feces, saliva
Corticosterone (17-deoxycortisol)	HMDB0001547	C_21_H_30_O_4_	346.214	[30]	Urine, blood
Cortisone	HMDB0002802	C_21_H_28_O_5_	360.194	[31]	Urine, blood
Dehydroascorbic acid	HMDB0001264	C_6_H_6_O_6_	174.016	[32,33]	Urine, blood
Deoxyguanosine	HMDB0000085	C_10_H_13_N_5_O_4_	267.097	No	Urine, blood, feces, saliva
Glutarylcarnitine	HMDB0013130	C_12_H_21_NO_6_	275.137	[27,34]	Urine, blood
Guanosine	HMDB0000133	C_10_H_13_N_5_O_5_	283.092	No	Urine, blood, feces, saliva
Indole-3-acetamide	HMDB0029739	C_10_H_10_N_2_O	174.079	No	Urine, blood
Methyl jasmonate	HMDB0036583	C_13_H_20_O_3_	224.141	No	Urine
5’-Methylthioadenosine	HMDB0001173	C_11_H_15_N_5_O_3_S	297.090	[29,35]	Urine, blood
Monoethyl malonic acid	HMDB0000576	C_5_H_8_O_4_	132.042	No	Blood
*N*-Acetyl-L-alanine	HMDB0000766	C_5_H_9_NO_3_	131.058	[29,36]	Urine, feces
*N*-Acetylleucine	HMDB0011756	C_8_H_15_NO_3_	173.105	No	Feces, saliva
*N*-Acetyl-L-methionine	HMDB0011745	C_7_H_13_NO_3_S	191.062	No	Feces, saliva
*N*-Acetyl-L-phenylalanine	HMDB0000512	C_11_H_13_NO_3_	207.090	[27]	Feces, saliva
Niacinamide	HMDB0001406	C_6_H_6_N_2_O	122.048	No	Urine, blood, feces, breast milk
*N*-methyl-L-glutamic Acid	HMDB0062660	C_6_H_11_NO_4_	161.069	No	Urine
l-Norleucine	HMDB0001645	C_6_H_13_NO_2_	131.095	No	Urine, blood, feces
l-Pipecolic acid	HMDB0000716	C_6_H_11_NO_2_	129.079	[37,38]	Blood, feces
Pyrrole-2-carboxylic acid	HMDB0004230	C_5_H_5_NO_2_	111.032	No	Urine, blood, feces
Sebacic acid	HMDB0000792	C_10_H_18_O_4_	202.121	No	Urine, blood, feces
Thyroxine	HMDB0000248	C_15_H_11_I_4_NO_4_	776.687	[39]	Urine, blood, saliva
trans-Aconitic acid	HMDB0000958	C_6_H_6_O_6_	174.016	No	Urine, blood

^1^ Monoisotopic masses with three decimals shown. ^2^ All metabolites not reported in CSF in the HMDB were further reviewed in the literature by manually searching PubMed (pubmed.gov) (accessed on 29 January 2020) and Google for “metabolite name” AND “cerebrospinal fluid”/”CSF”. Here we cite publications where the metabolites have previously been detected in CSF. ^3^ According to data in the HMDB.

**Table 2:** Age association in CSF from healthy subjects.

Metabolite	HMDB ID	q Value	p Age	p Gender	Coefficient for Age Association
Methylthioadenosine	HMDB0001173	0.06018	0.00153	N.S.	−0.02783
Pipecolate	HMDB0000716	0.06018	0.00168	N.S.	0.02495
Hippurate	HMDB0000714	0.06018	0.00238	N.S.	0.10119
Ketoleucine	HMDB0000695	0.0673	0.00394	N.S.	0.024
Isoleucine	HMDB0000172	0.0673	0.00507	N.S.	0.01664
Acetylcarnitine	HMDB0000201	0.0673	0.00531	N.S.	0.0179
Glutarylcarnitine	HMDB0013130	0.07023	0.00647	N.S.	0.00995
3-Methyladenine	HMDB0011600	0.07857	0.00827	N.S.	−0.02422
5-Hydroxytryptophan	HMDB0000472	0.08464	0.01002	0.04078	0.01451
Methionine	HMDB0000696	0.09243	0.01216	N.S.	0.02202

N.S.: Not significant.

**Table 3:** Demographic data of the studied healthy subjects.

Gender	*n*	Age in Years, Mean (±SD)	Age Range in Years
Female	16	47.3 (± 10.4)	30–68
Male	7	53.9 (± 10.3)	42–74

## Data Availability

Raw data and output of the metabolite identification are available on reasonable request to the corresponding author, K.K.

## Supplementary Materials

The following are available online at https://www.mdpi.com/2218-1989/11/2/126/s1, Table S1: CSF metabolites identified in the present study, Table S2: Gender and age of the individual participants.
