# Supplementary material for: Metabolomics of Cerebrospinal Fluid from Healthy Subjects Reveal Metabolites Associated with Ageing
Source: Metabolites. 2021 Feb 23;11(2):126. doi: 10.3390/metabo11020126 (PMC7927110; doi:10.3390/metabo11020126)
Supplement: Supplementary file 1 [file metabolites-11-00126-s001.pdf]

Supplementary Materials

Metabolomics of cerebrospinal fluid from healthy subjects reveal metabolites associated with ageing

Henrik Carlsson [1], Niclas Rollborn [1], Stephanie Herman [1], Eva Freyhult [1], Anders Svenningsson [2], Joachim Burman [3], Kim Kultima [1,*]

[1] Department of Medical Sciences, Clinical Chemistry, Uppsala University, Uppsala, Sweden
[2] Department of Clinical Sciences, Danderyd Hospital, Karolinska Institutet, Stockholm, Sweden
[3] Department of Neuroscience, Uppsala University, Uppsala, Sweden

* Correspondence: Kim.Kultima@medsci.uu.se

Supplementary Table 1 (Table S1): CSF metabolites identified in the present study

Metabolite[1]	HMDB ID	Formula	Mass[2] (Da)	Expected m/z	Observed m/z	Retention time (min)
1-Methyladenosine	HMDB0003331	C11H15N5O4	281.112	282.120	282.120	2.85
3-(2-Hydroxyphenyl)propanoic acid (melilotic acid)	HMDB0033752	C9H10O3	166.062	167.070	167.070	1.99
3-Methoxytyramine	HMDB0000022	C9H13NO2	167.094	168.102	168.102	1.19
3-Methoxytyrosine	HMDB0001434	C10H13NO4	211.084	212.092	212.092	1.26
3-Methyladenine	HMDB0011600	C6H7N5	149.070	150.077	150.077	0.84
4-Acetamidobutanoic acid	HMDB0003681	C6H11NO3	145.073	146.081	146.081	1.22
4-Guanidinobutanoic acid	HMDB0003464	C5H11N3O2	145.085	146.092	146.092	0.78
4-Hydroxybenzoic acid	HMDB0000500	C7H6O3	138.031	139.039	139.039	3.09
4-Methylcatechol	HMDB0000873	C7H8O2	124.052	125.060	125.060	1.99
4-Pyridoxic acid	HMDB0000017	C8H9NO4	183.053	184.060	184.058	0.84
5-Hydroxyindoleacetic acid	HMDB0000763	C10H9NO3	191.058	192.066	192.066	4.31
5-Hydroxy-L-tryptophan	HMDB0000472	C11H12N2O3	220.084	221.092	221.092	1.11
5-Methylcytosine	HMDB0002894	C5H7N3O	125.058	126.066	126.066	0.85
5'-Methylthioadenosine	HMDB0001173	C11H15N5O3S	297.089	298.097	298.097	5.32
Adenine	HMDB0000034	C5H5N5	135.054	136.062	136.062	0.80
Adenosine	HMDB0000050	C10H13N5O4	267.096	268.104	268.104	1.22
Aldosterone	HMDB0000037	C21H28O5	360.193	361.201	361.201	8.54
Allose	HMDB0001151	C6H12O6	180.063	179.056 [3]	179.055	0.61
Aminoadipic acid	HMDB0000510	C6H11NO4	161.068	162.076	162.076	0.90
Cortexolone (11-deoxycortisol)	HMDB0000015	C21H30O4	346.214	347.222	347.222	9.23
Corticosterone (17-deoxycortisol)	HMDB0001547	C21H30O4	346.214	347.222	347.222	9.23
Cortisol	HMDB0000063	C21H30O5	362.209	363.217	363.217	8.33
Cortisone	HMDB0002802	C21H28O5	360.193	361.201	361.201	8.54
Cyclic AMP	HMDB0000058	C10H12N5O6P	329.052	330.060	330.060	1.24

Dehydroascorbic acid	HMDB0001264	C6H6O6	174.016	175.024	175.024	0.84
Dehydroepiandrosterone	HMDB0000077	C19H28O2	288.208	289.216	289.214	10.19
Deoxyguanosine	HMDB0000085	C10H13N5O4	267.096	268.104	268.104	1.22
Dihydrouracil	HMDB0000076	C4H6N2O2	114.042	115.050	115.050	0.84
Glutarylcarnitine	HMDB0013130	C12H21NO6	275.136	276.144	276.144	1.19
Guanosine	HMDB0000133	C10H13N5O5	283.091	284.099	284.099	1.17
Hippuric acid	HMDB0000714	C9H9NO3	179.058	180.066	180.066	4.79
Hypoxanthine	HMDB0000157	C5H4N4O	136.038	137.046	137.046	0.84
Indole-3-acetamide	HMDB0029739	C10H10N2O	174.079	175.087	175.087	5.88
Inosine	HMDB0000195	C10H12N4O5	268.080	269.088	269.088	1.17
Ketoleucine	HMDB0000695	C6H10O3	130.062	131.070	131.070	3.60
Kynurenic acid	HMDB0000715	C10H7NO3	189.042	190.050	190.050	5.09
L-Acetylcarnitine	HMDB0000201	C9H17NO4	203.115	204.123	204.123	6.85
L-Glutamine	HMDB0000641	C5H10N2O3	146.069	147.076	147.076	0.69
L-Histidine	HMDB0000177	C6H9N3O2	155.069	156.077	156.077	0.69
L-Isoleucine	HMDB0000172	C6H13NO2	131.094	132.102	132.102	1.03
L-Kynurenine	HMDB0000684	C10H12N2O3	208.084	209.092	209.092	1.40
L-Leucine	HMDB0000687	C6H13NO2	131.094	132.102	132.102	1.03
L-Methionine	HMDB0000696	C5H11NO2S	149.051	150.058	150.058	0.80
L-Norleucine	HMDB0001645	C6H13NO2	131.094	132.102	132.102	1.03
L-Phenylalanine	HMDB0000159	C9H11NO2	165.078	166.086	166.086	1.63
L-Pipecolic acid	HMDB0000716	C6H11NO2	129.078	130.086	130.086	0.75
L-Tryptophan	HMDB0000929	C11H12N2O2	204.089	205.097	205.097	2.91
L-Tyrosine	HMDB0000158	C9H11NO3	181.073	182.081	182.081	0.89
Methyl jasmonate	HMDB0036583	C13H20O3	224.141	225.149	225.149	9.76
Monoethyl malonic acid	HMDB0000576	C5H8O4	132.042	133.050	133.050	0.89
N-Acetyl-L-alanine	HMDB0000766	C5H9NO3	131.058	132.066	132.066	0.98
N-Acetylleucine	HMDB0011756	C8H15NO3	173.105	174.112	174.113	6.44
N-Acetyl-L-methionine	HMDB0011745	C7H13NO3S	191.061	192.069	192.069	3.85
N-Acetyl-L-phenylalanine	HMDB0000512	C11H13NO3	207.089	208.097	208.097	6.70
Niacinamide	HMDB0001406	C6H6N2O	122.047	123.055	123.055	0.97
N-methyl-L-glutamic Acid	HMDB0062660	C6H11NO4	161.068	162.076	162.076	0.90
Pantothenic acid	HMDB0000210	C9H17NO5	219.110	220.118	220.118	2.92
Phenylacetic acid	HMDB0000209	C8H8O2	136.052	137.060	137.060	3.13
Pyrrole-2-carboxylic acid	HMDB0004230	C5H5NO2	111.031	112.039	112.040	2.28
Riboflavin	HMDB0000244	C17H20N4O6	376.138	377.146	377.143	6.43
S-Adenosylhomocysteine	HMDB0000939	C14H20N6O5S	384.121	385.129	385.129	1.06

Sebacic acid	HMDB0000792	C10H18O4	202.120	203.128	203.128	8.30
Testosterone	HMDB0000234	C19H28O2	288.208	289.216	289.214	10.19
Theobromine	HMDB0002825	C7H8N4O2	180.064	181.072	181.072	3.82
Thymine	HMDB0000262	C5H6N2O2	126.042	127.050	127.050	1.41
Thyroxine	HMDB0000248	C15H11I4NO4	776.686	777.694	777.695	9.47
trans-Aconitic acid	HMDB0000958	C6H6O6	174.016	175.024	175.024	0.84
Uracil	HMDB0000300	C4H4N2O2	112.027	113.035	113.035	0.91
Uridine	HMDB0000296	C9H12N2O6	244.069	245.077	245.074	0.68
Xanthine	HMDB0000292	C5H4N4O2	152.033	153.041	153.041	0.88

[1] All metabolites were identified in comparison with reference standards regarding m/z and retention time, and MS2 when available.

[2] Monoisotopic mass with three decimals shown.

[3] Allose was detected in the negative ionization mode, all other metabolites were detected in the positive ionization mode.

Supplementary Table 2 (Table S2): Gender and age of the individual participants

Participant No.	Gender Female (F)/Male (M)	Age in years
1	F	30
2	F	37
3	F	37
4	F	37
5	F	37
6	F	41
7	F	44
8	F	46
9	F	49
10	F	51
11	F	54
12	F	55
13	F	55
14	F	57
15	F	59
16	F	68
17	M	42
18	M	47
19	M	51
20	M	51
21	M	53
22	M	59
23	M	74
